# Chronic Exposure of Imidacloprid and Clothianidin Reduce Queen Survival, Foraging, and Nectar Storing in Colonies of *Bombus impatiens*

**DOI:** 10.1371/journal.pone.0091573

**Published:** 2014-03-18

**Authors:** Jamison Scholer, Vera Krischik

**Affiliations:** Department of Entomology, University of Minnesota, St. Paul, Minnesota, United States of America; University of Otago, New Zealand

## Abstract

In an 11-week greenhouse study, caged queenright colonies of *Bombus impatiens* Cresson, were fed treatments of 0 (0 ppb actual residue I, imidacloprid; C, clothianidin), 10 (14 I, 9 C), 20 (16 I, 17C), 50 (71 I, 39 C) and 100 (127 I, 76 C) ppb imidacloprid or clothianidin in sugar syrup (50%). These treatments overlapped the residue levels found in pollen and nectar of many crops and landscape plants, which have higher residue levels than seed-treated crops (less than 10 ppb, corn, canola and sunflower). At 6 weeks, queen mortality was significantly higher in 50 ppb and 100 ppb and by 11 weeks in 20 ppb–100 ppb neonicotinyl-treated colonies. The largest impact for both neonicotinyls starting at 20 (16 I, 17 C) ppb was the statistically significant reduction in queen survival (37% I, 56% C) ppb, worker movement, colony consumption, and colony weight compared to 0 ppb treatments. Bees at feeders flew back to the nest box so it appears that only a few workers were collecting syrup in the flight box and returning the syrup to the nest. The majority of the workers sat immobilized for weeks on the floor of the flight box without moving to fed at sugar syrup feeders. Neonicotinyl residues were lower in wax pots in the nest than in the sugar syrup that was provided. At 10 (14) ppb I and 50 (39) ppb C, fewer males were produced by the workers, but queens continued to invest in queen production which was similar among treatments. Feeding on imidacloprid and clothianidin can cause changes in behavior (reduced worker movement, consumption, wax pot production, and nectar storage) that result in detrimental effects on colonies (queen survival and colony weight). Wild bumblebees depending on foraging workers can be negatively impacted by chronic neonicotinyl exposure at 20 ppb.

## Introduction

Honey bees, bumblebees, and other native bees pollinate 30% of the plants that produce the vegetables, fruits, and nuts that we consume and more than 100 crops in North America require pollinators [1], [2]. Pollination contributes approximately $15 billion worth of additional crop yields [2], and wild bees contribute substantially to crop production [3].

In 2007, there were 49.5% fewer managed honey bee (*Apis mellifera*) colonies in North America than in 1961 [4]. Managed honey bee colony mortality has been estimated to be 30% since 2007 [5], [6]. Colony stressors include habitat loss, nutrient deficiencies, *Nosema* pathogens [7], [8], viruses [9], *Varroa* mites [6], pesticide exposure [10]–[12], interactions between *Nosema* and imidacloprid [13], [14], and *Nosema* and fipronil [15], [16]. Additionally, North American bumblebee species *Bombus occidentalis*, *B. pensylvanicus*, and B. *affinus* are in decline. These species had significantly higher *N. bombi* loads and lower genetic diversity compared to healthy populations [17], [18]. A combination of factors is most likely to contribute to bee losses [12], [19], [20].

The neonicotinyl insecticides, imidacloprid, thiamethoxam, clothianidin, and dinotefuran, were implicated in the decline of bees as they are systemic, accumulate in pollen and nectar, and are expressed for years from a single application [19], [21]–[25]. Neonicotinyls are applied in various ways (seed treatments, soil drenches, foliar sprays, irrigation systems, and tree injections) on agricultural and landscape plants. Most genetically modified crops (corn, canola, and soybeans) use seed treatments of imidacloprid (Gaucho), clothianidin (Poncho), or thiamethoxam (Crusier) [26]. The annual market for neonicotinyl insecticides is in the billions of dollars due to their low mammalian toxicity, systemic nature, and extended efficacy [27]. In the U.S., at least 58 million ha of the total 178 million ha of cropland are treated with over 907,185 kg of imidacloprid, clothianidin, and thiamethoxam [28]. In 2009 in Minnesota, most crops used seed treatments (corn, soybeans, potatoes, and canola) containing 21,212 kg of imidacloprid and 8,775 kg of clothianidin [29].

Residue levels of neonicotinyls in pollen and nectar differ depending on application method in crops and landscapes. Gaucho, an imidacloprid seed treatment of ≤1.0 mg/seed depending on the crop [30], [31], resulted in 4.4–7.6 ppb imidacloprid residue in canola pollen, 3 ppb in sunflower pollen, and 3.3 ppb in maize pollen [30], [32], [33]. An imidacloprid soil drench resulted in 122 ppb in pollen and 18 ppb in nectar of pumpkin [34] and 15 ppb in pollen and 10 ppb in nectar of squash [35].

Landscape applications of imidacloprid result in much higher levels of residue in nectar and pollen. A homeowner's formulation of imidacloprid, Bayer Advanced Tree and Shrub, or professional Marathon 1% G permits 270–300 mg to be applied to a 3 gallon pot, resulting in a 400 times higher application rate compared to Gaucho treated corn of 0.675 mg/seed. In USDA [25] research on tree injections and soil drenches, maple and horse chestnut flowers [32] were collected from trees that were trunk injected with imidacloprid 10–12 months earlier and residues of 130 ppb in 1 sample and 30–99 ppb in 5 samples were found. The report concluded that 130 ppb is in the range to cause mortality in bees. A soil injection around *Eucalyptus* trees resulted in 660 ppb imidacloprid in nectar which killed beneficial parasitic wasps [36]. Turf and white clover treated with clothianidin resulted in residues of 171 ppb in clover nectar. Colonies of *B. impatiens* did not avoid foraging on treated clover and showed reduced foraging activity and increased worker mortality in the hives within five days. Colonies showed a trend for fewer workers and males, no queen production, reduced number of wax pots, and reduced colony weight compared to controls [37]. Thus, the potential for neonicotinoid insecticides to impact bee health through chronic exposure may be currently underestimated as residue levels in agricultural and landscape plants are higher than reported for seed treatments.

Neonicotinyl insecticides are neurotoxins that affect mechanosensory stimuli, vision, olfaction, learning, and memory [38], [39]. Additionally, neonicotinoids bind to mushroom bodies in bee brains [39] which are particularly large in social bees compared to other insects, comprising over 40% of the neurons in the honeybee brains and less than 4% in *Drosophila* brains [40]. A 2.5 ppb imidacloprid or clothianidin dose affected Kenyon Cells (KC), by increasing excitability and inhibiting action potential firing, which impaired mushroom body function [41]. The effects of cholinergic pesticides on KCs are expected to lead to significant impairment of all cognitive functions that depend on this higher-order brain region, including multisensory integration, associative learning and memory, and spatial orientation.

Neonicotinoids are able to affect behavioral performance in honey bees [42]–[44]. Sublethal exposure of honey bees to neonicotinoids significantly impairs olfactory learning in laboratory-based studies [43], [44] and adversely affect navigation and foraging behavior in the field [19], [45]–[48]. Williamson and Wright [49] found that bees fed 13 ppb or 23 ppb imidacloprid were less likely to form long-term memory and had reduced learning. Eiri and Nieh [50] determined that foragers fed 0.21 ng/bee or 24 ppb imidacloprid produced significantly fewer waggle dance circuits (10.5- and 4.5-fold fewer for 50% and 30% sucrose solutions, respectively) 24 h later as compared to 0 ppb treatments. Waggle dancing can significantly increase colony food intake, and a sublethal dose may impair colony fitness.

Field studies on the effects of lower concentrations of neonicotinyl residue in pollen and nectar, similar to that found in seed treatments, usually showed no effects on colony health of honey bees and bumblebees. A study on queenright (containing the queen) colonies of *B. terrestris* for 4 weeks in the field near imidacloprid seed-treated sunflowers found no difference in worker or queen production [51]. Honey bees exposed for 4 months to flowering canola grown from clothianidin-treated seed (maximum of 2.24 ppb in nectar and 2.59 ppb in pollen) showed no differences in mortality, worker longevity, brood development, colony weight, and honey yields compare to controls [52].

However, some recent studies demonstrated that lower neonicotinyl concentrations alter bee colony health. Whitehorn et al. [53] showed that queenright colonies of *B. terrestris* fed 0.7 and 1.4 ppb imidacloprid in sugar syrup for 2 weeks in the lab and then monitored in the field for 6 weeks, could not recover from imidacloprid effects, colony weight was lower by 8% and 12% and queen production by 85% and 90%, respectively, compared to controls. Elston et al. [54] in laboratory studies demonstrated that *B. terretris* microcolonies fed 1 and 10 ppb thiamethoxam in sugar syrup for 4 weeks had reduced consumption of sugar syrup and production of wax storage pots.

Field and cage studies that exposed bees to higher amounts of neonicotinyl-treated sugar syrup have repeatedly shown reduction in colony health and bee foraging. In a 2-week study of queenright colonies of *B. terrestris* in flight cages within a greenhouse, bumblebees that were fed 10 and 20 ppb imidacloprid in sugar syrup had worker survival reduced by 62% and 95%, workers that would not forage, and no brood production compared to 0 and 2 ppb treatments [55]. A 4-week field study with queenright colonies of *B. terrestris* found that 10 ppb imidacloprid in sugar syrup reduced brood production by 22% and worker production by 27%, but did not increase queen or worker mortality or reduce colony weight. However, 50% of the workers did not return when foraging and were less efficient pollen collectors [47]. Foraging was reduced at 10 ppb imidacloprid for *B. terrestris* [47], [55] and 30 ppb imidacloprid for *B. impatiens* [56]. Honey bee foraging was reduced at 15 ppb imidacloprid [48], 5 ppb clothianidin [48], and 67 ppb thiamethoxam [46].

The objectives of this study were to investigate the effects of higher concentrations of imidacloprid and clothianidin, similar to those found in some crops and landscape plants, on individual behavior and colony health of the American bumblebee, *Bombus impatiens* Cresson by monitoring: 1) queen health (mortality and movement), 2) worker behavior (movement, colony, and bee consumption of sugar syrup), 3) colony health (colony weight, weight and number of wax pots containing stored sugar syrup, dead and alive brood, bees produced by caste, bees on nest, and worker bee weight).

## Materials and Methods

### Bumblebee colonies

Bumblebees used in this research were housed in clean cages and provided sufficient pollen and nectar for normal growth. An attached cage was used to permit foraging away from the nest. The hunidity and temperature of the ambient enviromnemnt was regulated to within the needs of the bees. At the end of the study, the entire nest was frozen before the colony was dissected.

We obtained commercially reared *Bombus impatiens* consisting of a queen and 30–50 workers (research grade A colonies approximately 1 month old) that were housed in a 25.4×22.9×12.7 cm plastic brood box (Koppert Biological Systems, Howell, MI). Colonies were fed Bee Happy sugar syrup (Koppert Biological Systems, Howell, MI) in the brood box. Once received, we assessed the colonies for the presence of the queen and number of workers by placing the plastic brood box into a 2-sleeve rearing cage (BioQuip Rancho Dominguez, CA) that was 35.6×35.6×61 cm under 2–100 watt red lights (Industrial Performance, Lenexa, KS) which made the bees more passive. In addition, 15 psi CO_2_ (20 pound carbon dioxide tank) was applied through a hose directly onto the colony, further reducing movement. We then removed all bees from the colony with a forceps (wide tip featherweight, BioQuip) and placed them into 30 mL wide mouth plastic vials and weighed the colony to the nearest gram (Taylor 3839 Glass Digital Diet Scale). The bees and nest were placed into a modified brood box with a Plexiglas lid (21.6×17.8×0.6 cm), which allowed for weekly photographs of the colony. The brood box was connected to a 29 cm square flight box (Bug Dorm 1, Bio Quip, Rancho Dominguez, CA) by a 1.9×30.5 cm plastic tube.

Colonies were established on benches in the greenhouse with temperature controlled to 22 C (Wadsworth Control System, STEP 50A) and humidity controlled to 60% (Aqua Fog Turbo XE). Additional environmental adjustments were made manually to temperature using fans to increase air circulation, and to humidity using a garden soaker hose placed underneath a greenhouse bench. Temperature and humidity were monitored with two data loggers (EL USB -1, Omega Engineering, Stamford, CT).

Supplemental pollen was collected from pollen traps on honey bee colonies on the St. Paul Campus of the University of Minnesota in summer 2010 and stored in a −20°C freezer. Pollen was mixed with Bee Happy to create a paste which could be molded into 7×1 cm rolls and coated with bees wax (Revlon Paraffin Spa RVS1213) and stored at −20°C. Pollen rolls were always available and were added every week to the floor of the brood box.

In the flight box, colonies were fed 50% sugar syrup from 118 ml round containers (Gladware) with a lid that was modified with a 2 cm hole through which a Koppert polyester wick was threaded. The syrup was always available and was replaced 3 times per week. Bees were fed untreated sugar syrup for 2 weeks prior to the start of the study.

### Experimental design

Colonies were provided imidacloprid or clothianidin in 50% sugar syrup for 5 treatments (0, 10, 20, 50, and 100 ppb) for 11 weeks. The experiment was performed twice for each neonicotinyl insecticide for a total of 8 colonies for each treatment (except 0 ppb clothianidin treatment had 9 colonies) (imidacloprid, July 6 to September 15, 2011 and September 14 to November 23, 2011 and clothianidin, January 18 to March 30, 2012 and March 12 to May 25, 2012).

Sugar syrup (50%) was made by adding granulated beet sugar (1000 g) (Cargill, Renville, MN) to 1000 mL deionized water. Analytical grade imidacloprid and clothianidin (Fischer Scientific, West Chester, PA, PS-2086, Lot no: 446-128B, 99.5 percent and PS-2261, Lot no:463-125A, 98.4 percent, respectively) were made into a 100,000 ppb stock solution by adding 0.02 grams (Sartorius ED323-CW milligram balance) into 200 mL of the sucrose solution (Fisher Scientific stirring plate 18×18 cm). Dilutions of 10, 20, 50, 100 ppb were made by pipetting 33.5, 67, 167.5, and 335 µL stock solutions (20–200 µL VWR Signature Ergonomic High Performance Single-Channel Variable Volume Pipettor) into bottles (PYREX Low Actinic 1 L Round Media Storage Bottles with red glass bottles to reduce light exposure) filled with 335 mL of 50% sugar syrup solution and stored at 5.5°C. Stock solutions were made every 3 weeks and sugar syrup solutions were made weekly.

### Residue analysis: Validation of imidacloprid and clothianidin in sugar syrup, pollen rolls, and wax syrup pots

Sugar syrup stock solutions were made continuously through the 11 weeks of the experiment, but syrup was analyzed for residue from one date for each replicate experiment (imidacloprid, August and October 2011; clothianidin, March and April 2012). For residue analysis, treated sugar syrup (0, 10, 20, 50, 100, and 100,000 ppb (stock)) samples were stored in 20 mL glass scintillation vials. Also, pollen (8 samples) used to make pollen rolls was stored for residue analysis.

For 3 dates, sugar syrup stored in wax pots was combined for three different colonies for each treatment and analyzed (imidacloprid, Sept and Nov 2011 (3 residue samples) and clothianidin, March and May 2011 (3 residue samples). Syrup extracted from all the wax pots in one colony was placed in 2×5 mm (2 ml) plastic microcentrifuge tubes and weighed. Both stock, pollen samples, and extracted syrup samples were kept at −80°C until shipped on dry ice to USDA, AMS, Gastonia, NC and analyzed for residue of imidacloprid and clothianidin parent compounds and metabolites and 4 fungicides using the standard USDA method (Table 1).

**Table 1:** Imidacloprid and clothianidin residue (ppb) in sugar syrup stock solutions (50%) from one sample in each replicate experiment and from stored syrup in wax pots (3 colonies mixed) from replicate 1(1 sample) and replicate 2 (2 samples) experiment, residue was determined by the standard USDA method, USDA, AMS, Gastonia, NC.

imidacloprid										
	Residue sugar syrup (ppb)				Residue stored syrup in wax pots at exp end (ppb)					
Planned trt	Aug 2011	Oct 2011	Mean residue	% diff plannedand residue	Sept 2011	Nov 2011	Nov 2011	Mean residue	% diff planned and residue	% diff residue trt/residue pot
**0 ppb**	0	0	0	0%	0	0	0	0	0%	**0%**
**10 ppb**	10	17	14	+40%	11	8	15	11	+10%	**−22%**
**20 ppb**	20	11	16	−20%	6	11	6	8	−60%	**−50%**
**50 ppb**	80	61	71	+42%	60	0	0	20	−40%	**−72%**
**100 ppb**	114	139	127	+27%	3	0	No sample	1	−99%	**−100%**
**100,000 ppb**	107,000	118,000	112,500	+13%	-	-	-	-	-	-

clothianidin										
	Residue sugar syrup (ppb)				Residue stored syrup in wax pots at exp end (ppb)					
Planned trt	March 2012	April 2012	Mean residue	% diff planned and residue	March 2012	May 2012	May 2012	Mean residue	% diff planned and residue	% diff residue trt/residue pot
**0 ppb**	0	0	0	0%	0	0	0	0	0%	**0%**
**10 ppb**	8	10	9	−10%	8	6	9	8	−20%	**−12%**
**20 ppb**	14	20	17	−15%	10	11	12	11	−45%	**−35%**
**50 ppb**	34	43	39	−22%	0	0	0	0	−100%	**−100%**
**100 ppb**	67	85	76	−24%	0	0	0	0	−100%	**−100%**
**100,000 ppb**	96,800	110,000	103,400	+3%	-	-	-	-	-	-

### Effect of chronic dose on queen mortality and queen and worker movement

Once a week, queen status (alive, dead, or absent) was recorded. Activity within each brood box was video recorded twice for 30 mins during weeks 4 and 8 (Bullet camera, Sony micro 550, NS 03-BU 4000HB, 12v, Recorder PV 1000, Lawmate, Stunt Camera, Grand Rapids, Michigan). From these videos, the movement of five workers and the queen were quantified by counting the number of seconds each bee moved in a total of 300 seconds. If the bee landed and remained motionless, then the seconds it was not moving were counted as 0. Bees that moved out of view were not used, so we were limited by the number of bees we could continuously monitor for 300 seconds. Bees were not marked, but only observed on the videos. Videos (30 mins) are stored on the computer in 3–10 min sections so it was easy to identify a single bee and not recount the bee.

### Effect of chronic dose on worker behavior

Syrup consumption per colony in the flight box was measured three times a week for each week (1–11 weeks) by pouring the remaining sugar syrup into a graduated cylinder. Individual bee consumption was estimated by dividing the mean weekly consumption by the number of bees on the nest.

### Effect of chronic dose on colony health

When the queen died or at week 11, colony weights were recorded and after, colonies were dissected. The number of wax pots containing sugar syrup was counted, and the syrup was transferred into 2 ml microcentrifuge tubes, weighed, and stored at −80C. Every week (0–11), a picture was taken of each colony (Nikon D100 camera, AF Nikon 28–105 mm macro lens) and pictures were analyzed for the number of wax pots containing sugar syrup and the number of bees on the nest (Microsoft Windows Paint, Windows 7 Enterprise). For each colony, the number of sugar syrup wax pots added during the experiment was determined by subtracting the number of pots at week 0 from week 11.

The brood (eggs, larvae and pupae) was counted and categorized as dead or alive according to color; brood was considered alive if white and firm and dead if discolored. The original queen and daughter queens were differentiated from workers by size [57]. Male bees were identified by the presence of a patch of yellow hair on the frons. At weeks 4, 6, and 8 bee weight was quantified by removing 20 foragers from the flight box of each colony. Bees were individually placed into 37 mL clear plastic solo cups on ice, individually weighed, painted on the dorsal thoracic sclerite, to ensure that a bee was not reweighted, and replaced into the flight box. Every other week dead bees were removed from the flight box, identified to caste, and frozen.

### Statistical analyses

Cumulative queen mortality, worker movement, and number of wax sugar syrup pots added were assessed with a Kruskal-Wallis, nonparametric Chi-Square test and a Wilcoxon nonparametric multiple comparison test [58]. Colony consumption, individual bee consumption, bees on nest, and bee weight were analyzed in ProcMixed [59] for treatment effects, week effects, and interaction effects, tested for homogeneity with a Levine test, transformed if needed, and assessed for treatment differences with a Tukey-Kramer multiple range test (MRT). If the Levene's test was significant after transformation, a Welch's test was used to correct for unequal variance. If there was a significant interaction in ProcMixed, then the data was analyzed with ANOVA's for all treatments by week. Colony weight, wax syrup pot weight, brood production (total, dead and alive), and bee caste production (worker, male, and queens) were tested for homogeneity with a Levine test, transformed if needed, and analyzed using ANOVA and a Tukey-Kramer MRT. If the Levene's test was significant after transformation, a Welch's test was used to correct for unequal variance [58].

## Results

### Residue analysis: Validation of imidacloprid and clothianidin in sugar syrup, pollen rolls, and wax syrup pots

When 8 pollen samples were tested for residue only 1 out of 8 samples had a 4 ppb imidacloprid residue and none of the samples had neonicotinyl metabolites or fungicides (carboxin, metalaxyl, tebuconazole, trifloxystrobin). Sugar syrup treatments were made from the 100,000 ppb stock solution every week or 11 times during each experiment using gravimetric and not molar methods. For all treatments and stock solutions, no neonicotinyl metabolites, and fungicides were found. Mean residues for stock solutions (100,000 ppb) for imidacloprid (I) and clothianidin (C) were slightly higher than what was planned (I, 13% greater and C, 3% greater). For imidacloprid, 1 of the 4 treatment residues is lower than the planned treatment and for clothianidin all 4 treatment residues are lower than the planned treatment (Table 1). The planned treatment is followed in parenthesis first by the actual mean residue that was measured and second by the percent difference between the planned treatment and actual residue (I: 0 ppb (0, 0%), 10 ppb (14, +40%), 20 ppb (16, −20%), 50 ppb (71, +42%), and 100 ppb (127, +27%); C: 0 ppb (0, 0%), 10 ppb (9, −10%), 20 ppb (17, −15%), 50 ppb (39, −22%), and 100 ppb (76, −24%)). In order to address the difference in planned treatments and actual residue measured, we added the actual residue mean in parentheses after the planned treatment in the abstract and discussed it in at the start of the discussion section.

For 3 sample dates (exp 1, n = 1 and exp 2, n = 2), sugar syrup stored in wax pots was combined for 3 different colonies for each treatment and analyzed. No metabolites of imidacloprid or clothianidin or any of the fungicides were found in the syrup stored in wax pots. The neonicotinyl residue in syrup stored in wax pots for 20–100 ppb imidacloprid and clothianidin treatments had considerably less residue than the planned treatments (Table 1). The planned treatment is followed in parentheses first by the actual residue in the wax pots that was measured and second by the percent difference between the treatment mean residue and the wax pot residue: I: 0 ppb (0, 0%), 10 ppb (11, −22%), 20 ppb (8, −50%), 50 ppb (20, −72%), and 100 ppb (1, −100%); C: 0 ppb (0, 0%), 10 ppb (8, −12%), 20 ppb (11, −35%), 50 ppb (0, −100%), and 100 ppb (0, −100%).

### Effect of chronic dose on queen mortality and queen movement

**Figure 1:**
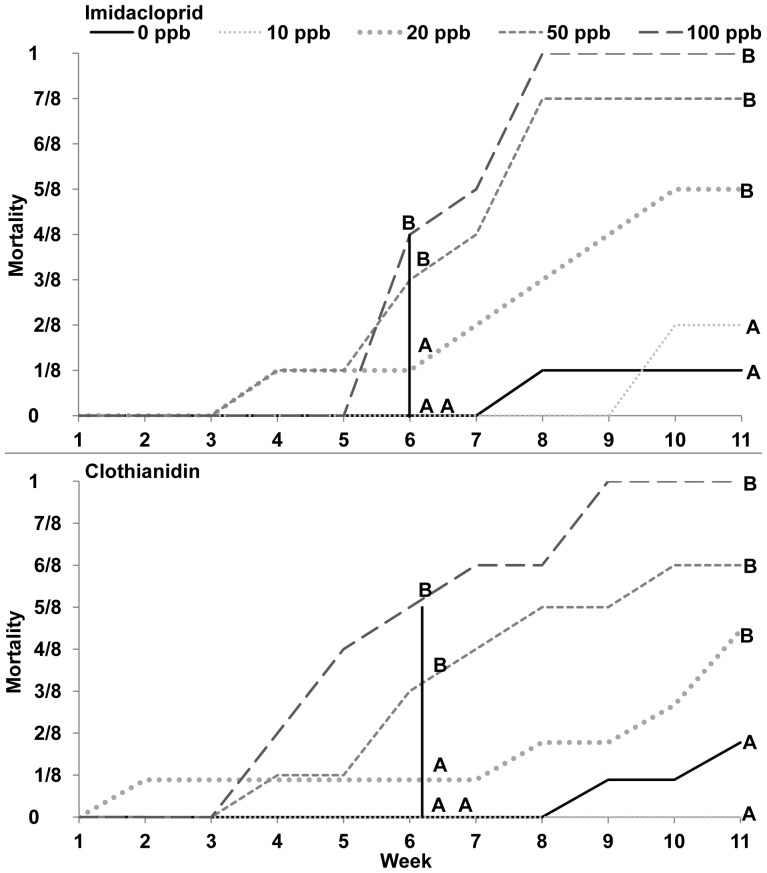
Queen mortality at weeks 1–11. **A,** Imidacloprid, Week 6: Chi-square test = 9.26, DF = 4, 235, p<0.055, week 11: Chi-square test = 75.49, DF = 4,435, p<0.001. **B,** Clothianidin, Week 6: Chi-square test = 22.87, DF = 4, 247, p<0.001, week 11: Chi-square test = 102.78, DF = 4, 457, p<0.001, Kruskal-Wallis, Wilcoxon Test.

Queens were never seen in the flight box at feeders, so queens fed on sugar syrup stored in wax pots. Imidacloprid and clothianidin treatments did not demonstrate immediate toxicity to queens, but by week 6 for both imidacloprid and clothianidin queen mortality was significantly lower in 0–20 ppb treatments compared to 50–100 ppb treatments. By week 11 for both imidacloprid and clothianidin, queen mortality was significantly lower in 0–10 ppb treatments compared to 20–100 ppb treatments (Figure 1, Kruskal-Wallis, Wilcoxon Test, SAS, JMP, 2012).

For both neonicotinyls, videos of queen movement revealed no significant differences among treatments (I, F = 1.70, DF = 4, 21, p = 0.188; C, F = 1.55, DF = 4, 6, p = 0.298, ANOVA, Tukey-Kramer, SAS JMP, 2012).

### Effect of chronic dose on worker behavior

Foraging bees went from the syrup feeders in the flight box through the tube to the nest box. However, most of the bees in the colony sat on the floor of flight box near the feeder, but were never seen moving to the feeders. The flight box bees stayed for weeks on the floor, were not observed to forage, moved slowly, and responded to probing with leg raising.

Videos of the nest box provided direct evidence that neonicotinyls reduced worker movement in the nest. We had to remove the 100 ppb treatment from the analysis as there were too few bees for which we could quantify movement. For imidacloprid, bees in 0 ppb moved significantly faster than those in 20 (47% slower) and 50 (59% slower) ppb treatments (0 ppb (178/300±20 sec), 10 ppb (126/300±9 sec), 20 ppb (94/300±240 sec), 50 ppb (73/300, ±21 sec)) (Chi-square test = 11.53, DF = 3, 25, p<0.0092, week 4 and 8). For clothianidin, bees in 0 ppb moved significantly faster than those in 20 ppb (32% slower) and 50 ppb (73% slower) treatments (0 ppb (117/300±23 sec), 10 ppb (82/300±12 sec), 20 ppb (79/300±19 sec), 50 ppb (32/300, ±9 sec)) (Chi-square test = 10.803, DF = 3, 28, p<0.0129, week 4 and 8).

**Figure 2:**
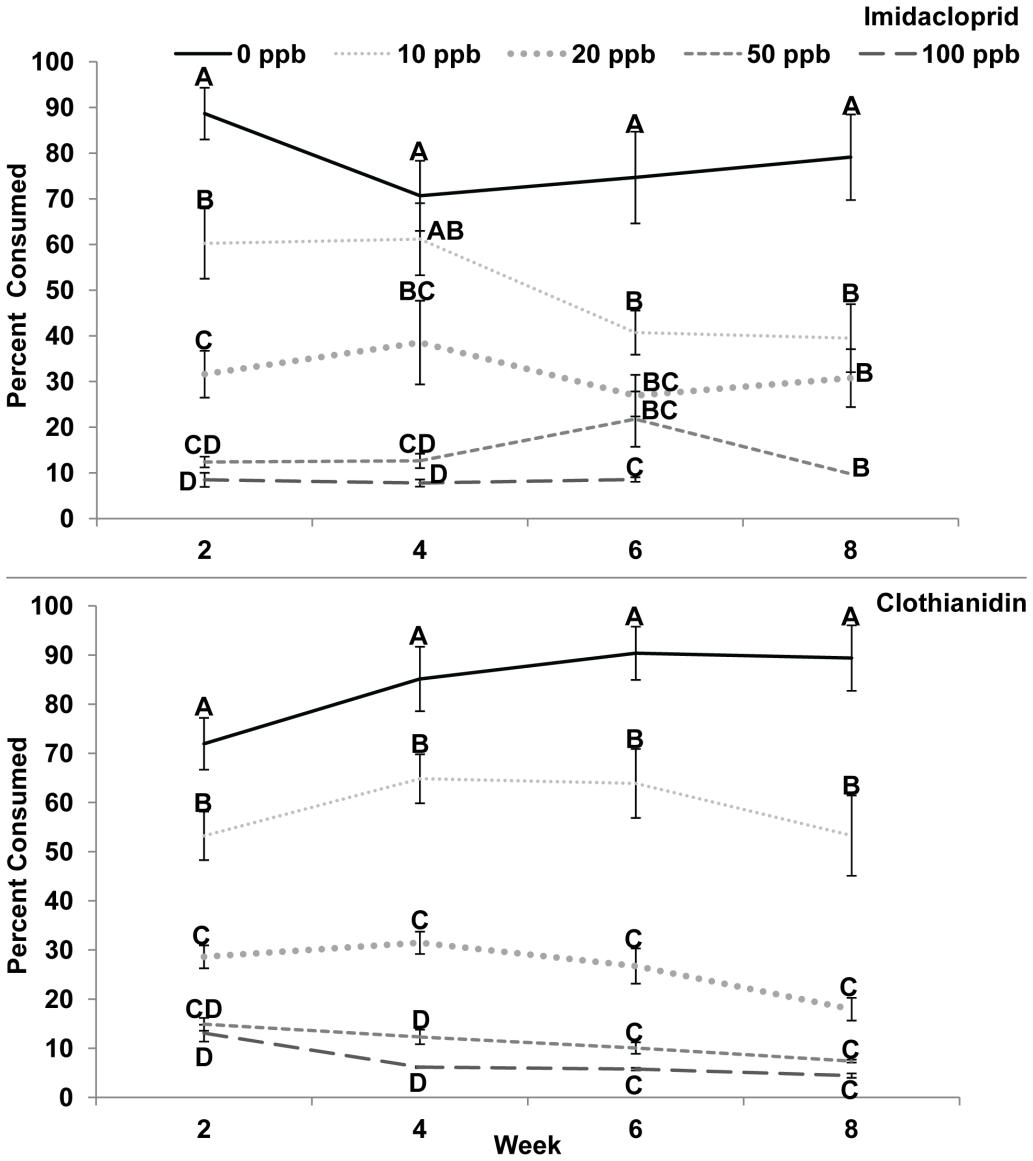
Colony consumption. **A,** Imidacloprid, Week 2: F = 52.51, DF = 4, 16, p<0.001, Week 4: F = 27.40, DF = 4, 14, p<0.001, Week 6: F = 22.61, DF = 4, 12, p<0.001, Week 8: F = 7.67, DF = 3, 17, p = 0.002. **B,** Clothianidin, Week 2: F = 42.05, DF = 4, 17, p<0.001, Week 4: F = 91.96, DF = 4, 14, p<0.001, Week 6: F = 42.77, DF = 4, 28, p<0.001, Week 8: F = 48.52, DF = 4, 8, p<0.001, ANOVA, Tukey-Kramer MRT by treatment for each week are on the figures, ProcMixed showed a significant interaction for imidacloprid and clothianidin, Table S1.

Colony consumption for imidacloprid and clothianidin, showed a significant interaction of week and treatment (Figure 2, Table S1, Proc Mixed, Tukey-Kramer, interaction effects, SAS, 2012) so the data were then analyzed by week for treatment using ANOVA and Tukey-Kramer, SAS, JMP, 2012). When colony consumption was analyzed by week, significantly more sugar syrup was consumed in weeks 2, 6, and 8 in 0 ppb compared to 10–100 ppb imidacloprid treatments and in weeks 2, 4, 6, and 8 in 10–100 ppb clothianidin treatments. In week 4 for imidacloprid, significantly more sugar syrup was consumed in 0 and 10 ppb compared to 20–100 ppb imidacloprid treatments (I: week 2, 10–100 ppb consumed 32%, 64%, 86%, and 90% less, respectively; week 4, 20–100 ppb consumed 45%, 82%, and 89% less, respectively; week 6, 10–100 ppb consumed 45%, 64%, 71%, and 89% less, respectively; week 8, 10–50 ppb consumed 50%, 61%, and 88% less, respectively; C: week 2, 10–100 ppb consumed 26%, 60%, 79%, and 82% less, respectively; week 4, 10–100 ppb consumed 24%, 63%, 86% and 94% less, respectively; week 6, 10–100 ppb consumed 29%, 70%, 89%, and 93% less, respectively; week 8, 10–100 ppb consumed 40%, 80%, 92%, and 95% less, respectively).

**Figure 3:**
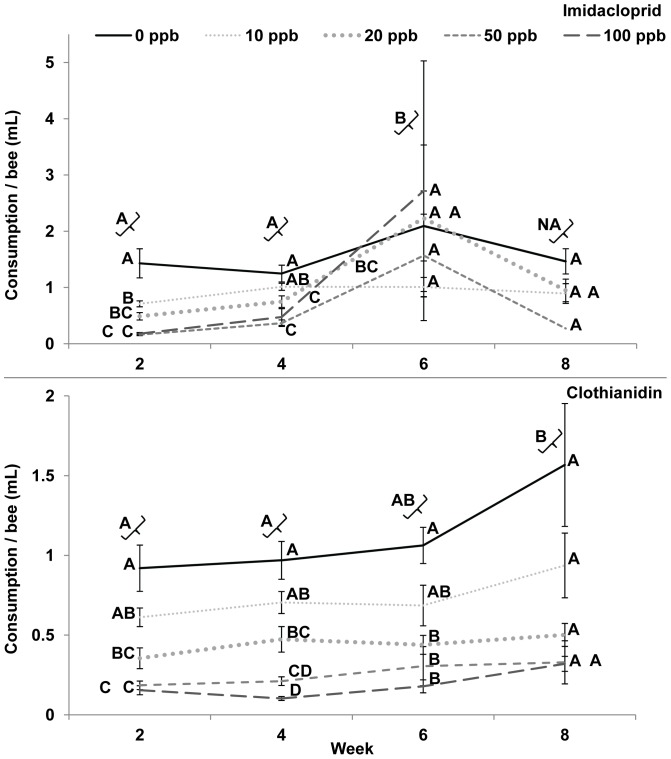
Bee consumption. **A,** Imidacloprid, Week 2: F = 30.97, DF = 4, 16, p<0.001, Week 4: F = 10.31, DF = 4, 33, p<0.001, Week 6: F = 0.89, DF = 4, 8, p = 0.513, Week 8: F = 2.51, DF = 3, 17, p = 0.093. **B,** Clothianidin, Week 2: F = 17.68, DF = 4, 17, p<0.001, Week 4: F = 32.73, DF = 4, 15, p<0.001, Week 6: F = 9.37, DF = 4, 28, p<0.001, Week 8: F = 4.32, DF = 4, 8, p = 0.035, ANOVA, Tukey-Kramer MRT by treatment for each week are on the figures to compare the 2 chemicals, but ProcMixed did not show a significant interaction for imidacloprid or clothianidin, Table S1.

Individual bee consumption was determined by dividing consumption per colony by the number of bees on the nest. For imidacloprid, individual bee consumption was not different between 0 ppb and 10–100 ppb treatments (Figure 3, Table S1, Proc Mixed, Tukey-Kramer, treatment effects, SAS, 2012). The amount (ml and g) that the bees consumed are presented in Table S2. When comparing weeks, week 6 had significantly more consumption compared to weeks 2 and 4 (Proc Mixed, Tukey-Kramer, week effects, SAS, 2012). However, when individual bee consumption was analyzed individually by week (ANOVA, Tukey-Kramer, SAS JMP, 2012), week 2 had significantly more sugar syrup consumed in 0 ppb compared to 10 ppb–100 ppb imidacloprid treatments (50%, 64%, 86%, and 86% less, respectively). In week 2 clothianidin treatments, had significantly more sugar syrup consumption in 0 ppb and 10 ppb treatments compared to 20–100 ppb treatments (61%, 80%, and 83% less, respectively). Week 4 had significantly more sugar syrup consumed in 0 compared to 20–100 ppb imidacloprid treatments (42%, 67%, and 100% less, respectively) and 20–100 ppb clothianidin treatments (51%, 78%, and 89% less, respectively). In week 6, ppb imidacloprid treatments were not statistically different. In week 6, clothianidin treatments were statistically different in 0 ppb compared to 20–100 ppb treatments (59%, 71%, and 83% less, respectively). In week 8 there was no statistical difference among treatments. The ng consumed by a bee for each treatment by week was reported in Table S2.

### Effect of chronic dose on colony health

**Figure 4:**
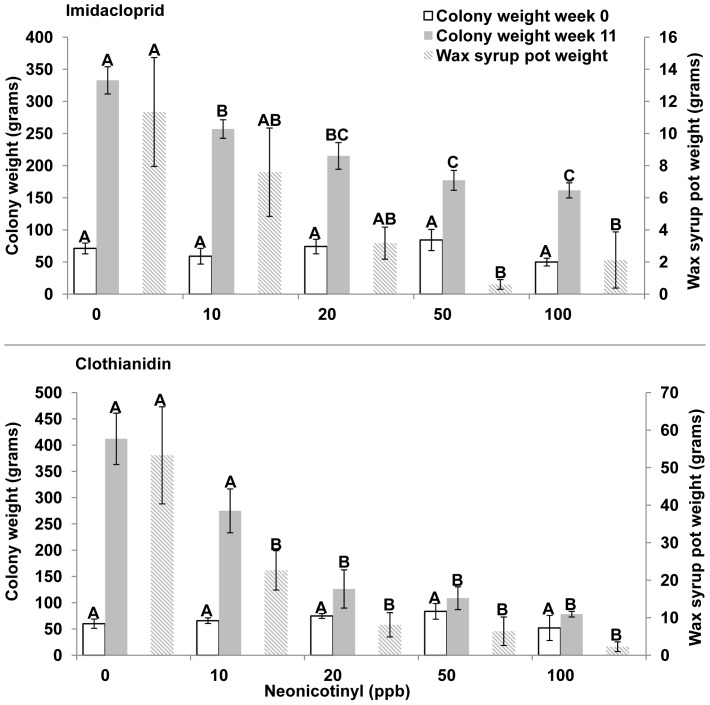
Colony weight and syrup weight in wax pots. **A,** Imidacloprid, colony weight, Week 0: F = 1.84, DF = 4, 16, p = 0.170, Week 11: F = 16.20, DF = 4, 35, p<0.001; syrup weight, Week 11: F = 4.83, DF = 4, 15, p = 0.011. **B,** Clothianidin, colony weight, Week 0: F = 0.87, DF = 4, 37, p = 0.492, Week 11: F = 16.10, DF = 4, 37, p<0.001; syrup weight Week 11: F = 6.83, DF = 4, 16, p = 0.002, ANOVA, Tukey-Kramer MRT.

Colony weight at week 0 was the same for all treatments of imidacloprid or clothianidin. At week 11, colony weight was significantly greater in 0 ppb (350 g) compared to 10–100 ppb imidacloprid treatments (23%, 35%, 47%, and 51% less, respectively) and was significantly greater in 0 ppb (412 g) and 10 ppb (275 g) compared to 20 100 ppb clothianidin treatments (69%, 74%, and 81% less, respectively) (Figure 4, ANOVA, Tukey-Kramer, SAS JMP, 2012).

The weight of syrup in wax pots in imidacloprid treatments was significantly greater in 0 ppb (11.3 g), 10 ppb (7.6 g), and 20 ppb (3.2 g) compared to 50 ppb (2.0 g, 95% less) and 100 ppb (1.0 g, 81% less) treatments. The weight of syrup in wax syrup pots was significantly greater in clothianidin treatments in 0 ppb (53.3 g) compared to 10 ppb (22.6 g, 58% less), 20 ppb (8.1 g, 85% less), 50 ppb (7.3 g, 86% less) and 100 ppb (2.3, 96% less) (Figure 4, ANOVA, Tukey-Kramer, SAS JMP, 2012).

**Figure 5:**
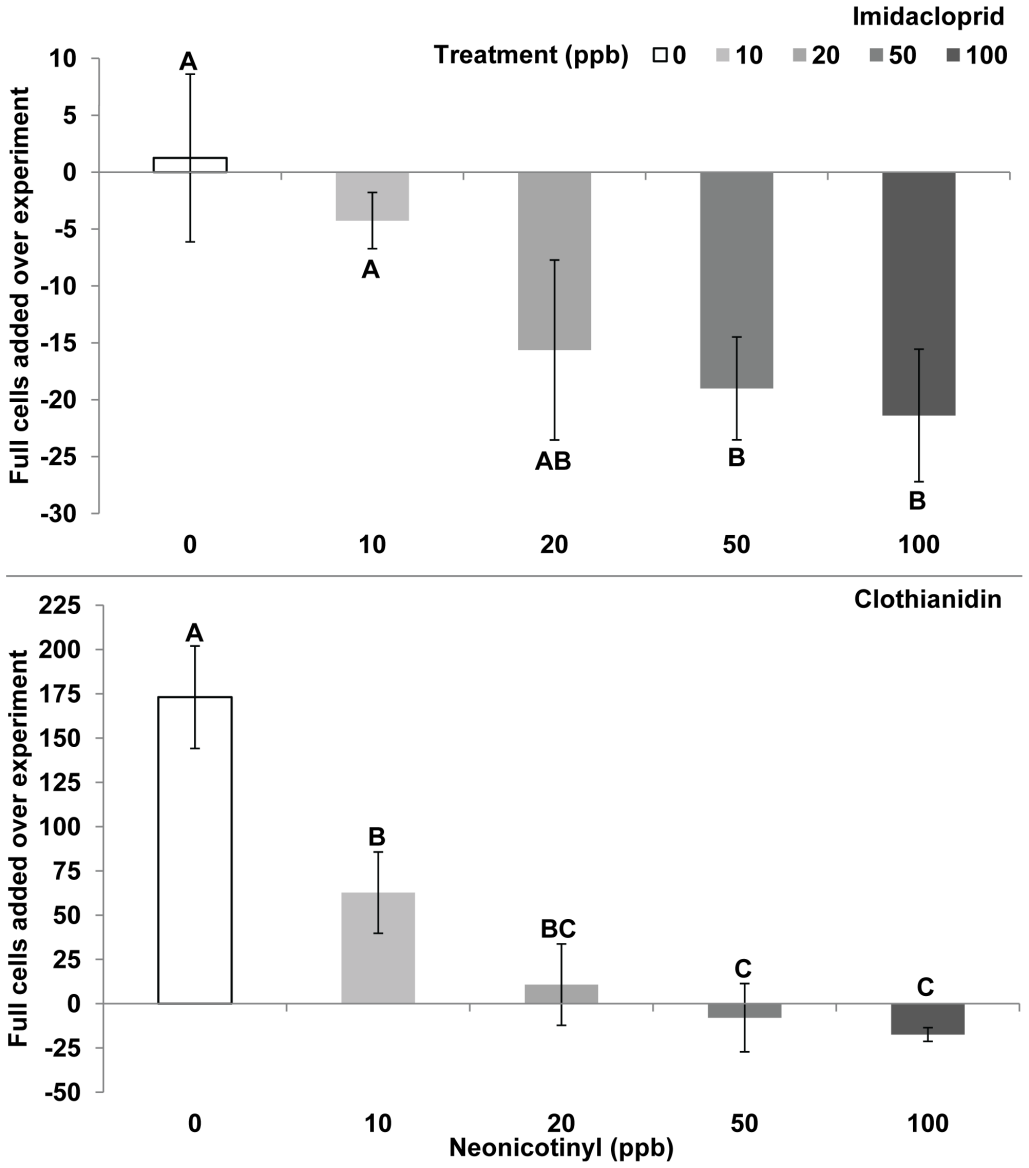
Wax syrup pots added. **A,** Imidacloprid, Chi-square test = 10.23, DF = 4, p = 0.0368. **B,** Clothianidin, Chi-square test, F = 21.54, DF = 4, p<0.0002, Kruskal-Wallis, Wilcoxon Test.

For the 0 ppb imidacloprid treatment the number of wax pots at the start of the experiment was 21 pots and 1.2 pots were added. For imidacloprid treatments the number of wax syrup pots added was significantly greater in 0 ppb (+1 pot) compared to 50 ppb (−19 pots, 2,000% less) and 100 ppb (−21 pots, 2,200% less) treatments. For the 0 ppb clothianidin treatment, the number of wax pots at the start of the experiment was 36 pots and 173 pots were added. For clothianidin treatments the number of stored syrup pots added was significantly greater in 0 ppb (173 pots) compared to 10 ppb (63 pots, 64% less), 20 ppb (11 pots, 94% less), 50 ppb (−8 pots, 105% less) and 100 ppb (−17 pots, 110% less) treatments (Figure 5, Kruskal-Wallis, Wilcoxon Test, SAS JMP, 2012).

As treatment concentration increased for both imidacloprid and clothianidin, lower residue was found in the sugar syrup in wax pots. Treatments of 50–100 ppb imidacloprid and clothianidin had 72–100% less residue than the concentration in the syrup the bees were consuming, which indicated that sugar syrup was not being stored and supported the data that wax pots numbers and weighs decreased. For imidacloprid, 0 ppb contained no residue, 10 ppb contained 11 ppb or 22% less residue, 20 ppb contained 8 ppb or 50% less residue, 50 ppb contained 20 ppb or 72% less residue, and 100 ppb contained 1 ppb or 100% less residue. For clothianidin, 0 ppb contained no residue, 10 ppb contained 8 ppb or 12% less residue, 20 ppb contained 11 ppb or 35% less residue, 50 ppb contained 0 ppb or 100% less residue, and 100 ppb contained 0 ppb or 100% less residue (Table 1).

**Figure 6:**
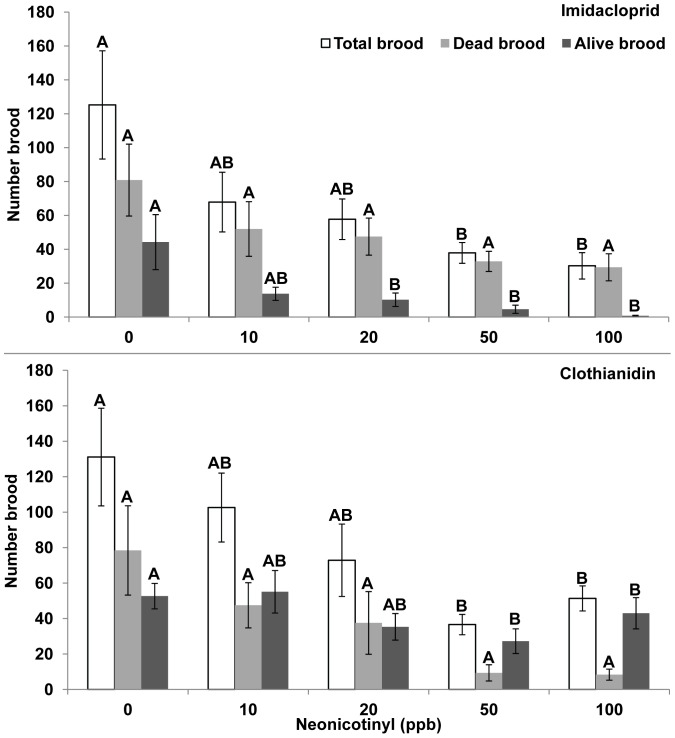
Total, dead, and alive brood. **A,** Imidacloprid, Week 11: Total Brood: F = 2.99, DF = 4, 17, p = 0.049, Dead Brood: F = 1.67, DF = 4, 17, p = 0.205, Alive Brood: F = 5.74, DF = 4, 14, p = 0.006. **B,** Clothianidin, Week 11: Total Brood: F = 4.16, DF = 4,37, p = 0.007, Dead Brood: F = 1.83, DF = 4,37, p = 0.144, Alive Brood: F = 4.13, DF = 4,17, p = 0.016, ANOVA, Tukey-Kramer MRT.

Neither neonicotinyl demonstrated toxicity to brood, as dead brood was not significantly different among treatments. However, at week 11 the amount of alive brood was significantly greater in 0 ppb compared to 20–100 ppb imidacloprid treatments and 50–100 ppb clothianidin treatments, reflecting premature queen mortality. Total brood (dead and alive) for both imidacloprid and clothianidin was significantly greater in 0 ppb compared to 50 and 100 ppb as a result of less alive brood by week 11 (Figure 6, ANOVA, Tukey-Kramer, SAS JMP, 2012).

**Figure 7:**
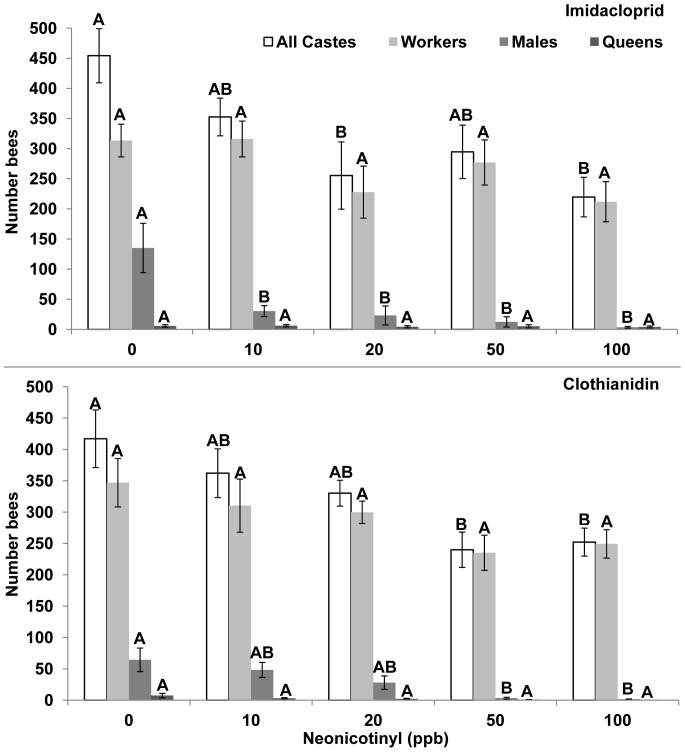
Worker, male, and queen production. **A,** Imidacloprid, Week 11: All Castes: F = 4.62, DF = 4, 35, p = 0.004, Workers: F = 1.92, DF = 4, 35, p = 0.129, Males: F = 4.59, DF = 4, 14, p = 0.014, Queens: F = 0.19, DF = 4, 35, p = 0.945. **B,** Clothianidin, Week 11: All Castes: F = 5.12, DF = 4, 37, p = 0.002, Workers: F = 2.15, DF = 4, 37, p = 0.094, Males: F = 7.44, DF = 4, 16, p = 0.002, Queens: F = 2.23, DF = 4, 37, p = 0.085, ANOVA, Tukey-Kramer MRT.

For both neonicotinyls, daughter queen production were not significantly different among treatments for either imidacloprid (0–100 ppb produced 5.7, 6.1, 4.3, 5.1; and 4.1 queens) or clothianidin (0–100 ppb produced 7.4, 3.1, 2.2, 1.1, and 1 queens). Although for clothianidin, there was a trend for fewer queens produced in 10–100 ppb treatments compared to 0 ppb treatments. The number of workers produced was not significantly different among treatments. However the mean number of males produced in imidacloprid treatments was significantly greater in 0 ppb compared to 10–100 ppb treatments (0–100 ppb produced 135; 30; 23; 50, 13; and 4 males). For clothianidin treatments the mean number of males produced was significantly greater in 0 compared to 50–100 ppb treatments (0–100 ppb produced 64; 48; 28; 3; and 2 males) (Figure 7, ANOVA, Tukey-Kramer, SAS JMP, 2012).

For imidacloprid, the number of bees on the nest (cast was not visible on pictures) was not significantly different among treatments, but significantly decreased from weeks 2–6 (Figure S1, Table S1, Proc Mixed, Tukey-Kramer, week effects, SAS, 2012). However when weeks were individually analyzed, week 4 and 6 had significantly more bees on the nest in 0 ppb compared to 100 ppb treatments (0 ppb (wk 4, 48.1±8 9.0, wk 6, 41.0 8.7), 10 ppb (wk 4, 46.1±5.8, wk 6, 32.6±4.5), 20 ppb (wk 4, 37.4±7.0, wk 6, 22.5±6.3), 50 ppb (wk 4, 28.9±5.0, wk 6, 17.6±4.1), and 100 ppb (wk 4, 19.1±3.7, wk 6, 8.5±5.3) (Figure S1, Table S1, ANOVA, Tukey-Kramer, SAS JMP, 2012).

For clothianidin, the numbers of bees on nest when analyzed showed a significant interaction of week and treatment (Figure S1, Table S1, Proc Mixed, Tukey-Kramer, interaction effects, SAS, 2012). However, when weeks were individually analyzed only at week 6, were significantly more bees on the nest in 0 and 10 ppb treatments compared 50 ppb and 100 ppb treatments (0 ppb (72.4±11.1), 10 ppb (79.6±10.1), 20 ppb (51.6±8.5) and 50 ppb (33.8±5.9), and 100 ppb (23.0±4.0) (Figure S1, Table S1, ANOVA, Tukey-Kramer, SAS JMP, 2012).

For imidacloprid, bee weight was not significantly different among treatments and bee weight decreased significantly between weeks 6 and 8 (0 ppb (wk 6, 0.15±0.01, wk 8, 0.13±0.01), 10 ppb (wk 6, 0.15±0.01, wk 8, 0.11±0.01), 20 ppb (wk 6, 0.13±0.1, wk 8, 0.11±0.01), 50 ppb (wk 6, 0.14±0.01, wk 8, 0.10±0.01), and 100 ppb (wk 6, 0.14±0.03, wk 8, 0.07±0.02) (Table S1, Proc Mixed, Tukey-Kramer, week effects, SAS, 2012).

For clothianidin, bee weight was significantly different between the 0 and 20 ppb treatments and bee weight decreased significantly between week 4 and 6 (0 ppb (wk 4, 0.12±0.01, wk 6, 0.10±0.01), 10 ppb (wk 4, 0.13±0.02, wk 6, 0.14±0.01), 20 ppb (wk 4, 0.16±0.01, wk 6, 0.15±0.01), 50 ppb (wk, 4 0.19±0.02,wk 6, 0.12±0.02), and 100 ppb (wk 4, 0.13±0.03, wk 6, 0.09±0.01) (Table S1, Proc Mixed, Tukey-Kramer, treatment and week effects, SAS, 2012).

## Discussion

Very few papers confirm treatment residues with analytical methods, however in this research we did and the planned treatments were slightly different in concentration than the actual residue (Table 1). In retrospect, 2 samples (1 for each replicate experiment for each treatment) to verify residue were too small a number and it would have been better to collect 1 sample each week for the 11 week study to determine the residue. We speculate that with more residue samples, the mean of the residue concentration would be closer to the planned treatment concentration. The mean treatment residues do not overlap, which supports that the treatments were relatively different and provided a concentration gradient. The planned treatments were chosen to represent a range of potential residue found in pollen and nectar from crops and landscape plants. Neonicotinyl treatments used in this study ranged from 10 ppb the highest amount found in seed-treatments to 100 ppb levels found in landscape plants (Table 1). Our highest concentration of 100 ppb imidacloprid was below the estimated oral LC50 for honey bees of 185 ppb [60] or 192 ppb [61]. In this study, both of these neonicotinyls had similar toxicity, as expected by their similar acute oral LD50s: for imidacloprid 4–40 ng/bee for honey bees [33], [62] and 2 ng/bee for bumblebees [63] and for clothianidin 22 ng/bee for honey bees [33], [45].

Our study demonstrated that 20 ppb imidacloprid or clothianidin fed to queenright colonies of *B. impatiens* for 11 weeks increased queen mortality, reduced colony consumption, and colony weight. Starting at 6 weeks, queen mortality was significantly higher in 50–100 ppb imidacloprid- and clothianidin- treated colonies and by 11 weeks in 20 ppb–100 ppb imidacloprid- and clothianidin- treated colonies. Colony consumption for imidacloprid and clothianidin was significantly less at 20–100 ppb. The weight of syrup in wax pots and number of wax pots added was significantly less at 50–100 ppb imidacloprid treatments and at 10–100 ppb clothianidin treatments. Colony weight was significantly less at 10–100 ppb imidacloprid treatments and 20–100 ppb clothianidin treatments. In both imidacloprid and clothianidin, the residue in wax syrup pots for 50 and 100 ppb was 72–100% less residue indicating that syrup was not being returned to the pots (Table 1). Neither neonicotinyl decreased worker and queen production, but male production was reduced at 10–100 ppb imidacloprid treatments and 50–100 ppb clothianidin treatments. There were not differences in number of dead brood, indicating imidacloprid and clothianidin were not toxic to young bees, unless the brood was feeding on untreated syrup stored before the start of the experiment. Significantly more total brood production was a result of more alive brood, since queen mortality occurred earlier in 50–100 ppb treatments.

Our study demonstrated that both imidacloprid and clothianidin caused significant mortality in 20–100 ppb treatments, which is important data as there is little published data on the effects of neonicotinyl insecticides on queen bumblebees, since most studies use queenless microcolonies containing only workers. Our study did not find any effects of either neonicotinyl on worker numbers, although other studies have shown reduction in worker numbers starting at 10 ppb. An 11 week study on *B. terrestris* in queenless microcolonies found that worker mortality was 0% at 0 and 10 ppb, 50% at 20 ppb, and 100% at 200 ppb imidacloprid. Thiamethoxam at 0 ppb showed 0% worker mortality compared to 85% mortality at 100 ppb [55]. Laboratory feeding tests with *B. terrestris* at 2 doses, 10 ppb in sugar syrup and 6 ppb in pollen, and 25 ppb in sugar syrup and 16 ppb in pollen, found that imidacloprid significantly reduced worker survival by 10% in 4 weeks [66]. In 76 queenless microcolonies of *B. terrestris* exposed to imidacloprid at 10 doses from 0.08 ppb to 125 ppb, only one worker died at 125 ppb [65].

In our study daughter queen production was not significantly different for imidacloprid treatments (0–100 ppb produced 5.7, 6.1, 4.3, 5.1; and 4.1 queens) although for clothianidin there was a nonsignificant trend for fewer queens produced in 10–100 ppb treatments (7.4, 3.1, 2.2, 1.1, and 1 queens). However, the mean number of males produced was significantly lower in 10–100 ppb imidacloprid treatments (0–100 ppb produced 135, 30, 23, 13, 4 males, respectively) and 50 and 100 ppb clothianidin treatments (0 100 ppb produced 64, 48, 28, 3, 2 males, respectively). Others have considered a link between neonicotinyl insecticides and male production. Laycock et al. [65] using microcolonies found that male production was negatively dose-dependent (0 to 125 ppb imidacloprid, 42% less males produced at 1.27 ppb), but reduction in ovary development was found only at the highest dosage of 125 ppb imidacloprid. However, queenless microcolonies that consumed more syrup and pollen produced more brood. Higher imidacloprid doses reduced pollen and syrup feeding, so lack of nutrition was suspected as the mechanism behind reduced male production by workers [65]. Another greenhouse study on queenless microcolonies of *B. terrestris* found similar effects of decreased feeding, increased foraging time, and decreased male production with neonicotinyl insecticides. Queenless microcolonies fed 0, 10, 20 and 200 ppb imidacloprid had lower male production at 20 ppb, workers feed and foraged less, and it took longer to fly between food and the nest [55]. Another greenhouse study found that queenless microcolonies of *B. impatiens* fed 19 ppb imidacloprid-treated pollen consumed significantly less pollen, had shorter worker longevity, and produced no males compared to 0 ppb [73].

Colony health was quantified by the weight and number of wax pots containing stored sugar syrup and colony weight. In 0 ppb treatments, bees secreted wax and added it to the colony nest structure to make new sugar syrup pots, gathered sugar syrup from small containers in the flight box, and filled the wax pots with sugar syrup, thereby increasing the number of stored syrup pots, the weight of the syrup wax pots, and the entire colony weight. In higher neonicotinyl treatments, nest bees emptied the storage pots filled prior to treatment and did not re-fill old pots. This is further supported by the reduction in colony consumption at 10–100 ppb. Lack of new syrup storage is also supported by the residue analysis data. In both imidacloprid and clothianidin, the residue in wax syrup pots for 50–100 ppb was 72–100% less residue than the concentration in the syrup the bees were consuming indicating that syrup was not being returned to the pots in 50–100 ppb treatments (Table 1).

Other studies demonstrated a link between neonicotinyl consumption and colony weight. . Our results are similar to Elston et al. [54] that demonstrated *B. terretris* microcolonies fed 1 and 10 ppb thiamethoxam compared to controls had reduced consumption of sugar syrup (1 ppb, 1.54 g less and 10 ppb, 1.16 g less) and production of wax pots (1 ppb, 10% less and 10 ppb, 100% less) in 28 days. Queenright colonies of *B. terrestris* provided 6 ppb imidacloprid pollen plus 0.7 ppb imidacloprid nectar and double the dose for 2 weeks then placed in the field for 6 weeks had reduced colony weights of 8% and 12% and reduced daughter queen production of 85% and 90%, respectively [53]. Queenright colonies of *B. impatiens* did not avoid foraging on clothianidin-treated clover (171 ppb nectar) and showed reduced foraging activity and increased worker mortality in the hives within 5 days. Colonies showed a trend for fewer workers and males, no queen production, reduced number of wax pots, and reduced colony weight compared to controls [37]. Reduced colony weight is related to worker foraging and behavior.

Videos inside nest boxes showed that nest bees moved faster in 0 ppb compared to 20 and 50 ppb imidacloprid and clothianidin treatments. We speculate that nest bees that went into foraging boxes to collect neonicotinyl-treated sugar syrup were impaired as a result of ingesting and detoxifying the insecticides, fed less, moved less, and returned less syrup to the colony. Older nest bees did not return to the hive but sat on the floor of the nest box for weeks, not feeding, probably physiologically impaired as a result of chronically consuming neonicotinyl-treated sugar syrup prior to their resting stupor. When a bee consumes a neonicotinyl, symptoms such as knockdown, trembling, and uncoordinated and hyperactive movement occur quickly, before the insecticide is detoxified in 6 hours and the bee recovers or dies [67]–[70]. Thus, bees can recover from chronic, sublethal doses of neonicotinyl insecticides, feed, and start the syndrome again. For honey bees, an imidacloprid dose of 5 ng/bee was transformed in 24 hrs into the metabolites 5-hydroxy-imidacloprid and olefin, before being detoxified by the bee [67]. For bumblebees, an imidacloprid dose of 4.8 ng/bee was transformed quickly and metabolites were not detected in the bee [66].

We demonstrated reduction in movement starting at 20 ppb, colony consumption at 20 ppb, and storage pot weight at 50 ppb imidacloprid and 10 ppb clothianidin treatments. Reduced movement, consumption, and storage are factors associated with foraging. Many other studies have demonstrated that neonicotinyls reduce foraging. Foraging was reduced at 10 ppb imidacloprid for *B. terrestris* [47], [55] and 30 ppb imidacloprid for *B. impatiens* [56]. Honey bee foraging was reduced at 15 ppb imidacloprid [48], 5 ppb clothianidin [48], and 67 ppb thiamethoxam [46]. Imidacloprid at 5 ng/bee ( = 50 ppb) impaired the ability of bumblebee foragers to orient to landmarks and return to their nests in the field [64]. Imidacloprid and clothianidin at 2.5 ppb impaired mushroom body function that can lead to significant impairment of all cognitive functions associated with foraging that depend on this higher-order brain region, including multisensory integration, associative learning and memory, and spatial orientation [41].

Similar to our foraging results, a greenhouse cage study on queenright microcolonies of *B. terrestris* provided imidacloprid-treated sugar syrup found that bees were lethargic and spent less time foraging. At 20 ppb, the workers stayed near the nectar and pollen, were apathetic, did not move or forage, and eventually died by the food, whereas at 10 ppb all dead workers were found inside the nests and at 2 ppb, there was no reduction in worker movement and no mortality [55]. Greenhouse cage studies with *B. terrestris* fed flowers from cucumbers sprayed with the 4 mg/sgft of imidacloprid found that the bees stopped foraging and sat still for several hours and recovered or died [71]. In greenhouse cage studies with *B. impatiens* workers fed 30 ppb imidacloprid in 30% sugar syrup, workers spent 43% more time accessing flowers and 28% more time foraging compared to 0 and 7 ppb [56]. Tunnel studies with imidacloprid-treated sugar syrup at 6 ppb found reduced number of active honey bees, resulting in more inactive bees sitting at the feeders [72].

The reduction in bumblebee foraging due to neonicotinyl treated sugar syrup found in greenhouse studies was supported by field studies. Gill et al. [47] found that bees fitted with RFID (radio frequency identification tags) and fed 10 ppb imidacloprid in sugar syrup for 4 weeks had significantly more workers (50%) that did not return to the colony. Worker foraging performance, particularly pollen collecting efficiency, was significantly reduced which led to increased colony demand for food as shown by increased worker recruitment to forage and less time spend on brood care. Averill [64] found that imidacloprid at 5 ng/bee (50 ppb) impaired the ability of foragers to orient to landmarks when displaced away from their nests in the field. In the field, imidacloprid seed-treated sunflowers reduced *B. terretris* forager return by 10% (33% treated and 23% 0 ppb), although residue in pollen and nectar were unknown [51].

Our data provide mechanisms that link foraging behavior and colony health and offer strong support that chronic exposure to imidacloprid or clothianidin starting at 20 ppb significantly reduced colony health (lower colony weight, less wax pots added, and higher queen mortality) as a result of decreased worker foraging (movement, consumption, and storage of syrup). In 12 research papers discussed above sublethal, chronic effects on foraging were found. Since most studies show reduction in foraging behavior below 10 ppb and residues in crop and landscape flowers are higher than 10 ppb, bees are likely to be experiencing chronic, sublethal doses that will reduce navigation and foraging and lead to colony failure. Social bee colonies, such as bumblebees and honey bees use division of labor and rely on foragers to return nectar and pollen to the hive for the queen, nest bees, and brood. Native, annual bee colonies and queens in spring and fall are even more vulnerable to neonicotinyl insecticides since the solitary queens can be impaired when foraging and instead of workers not returning to the nest the result will be the death of the queen and loss of future generations. The collective research data provide support that bee foraging is reduced by neonicotinyl insecticides and continued indiscriminate use of systemic, neonicotinyl insecticides, that last from a single application for months to years in pollen and nectar, will reduce bee numbers and reduce seed and fruit production, resulting in dramatic ecosystem consequences.

## Supporting Information

Figure S1 **Bees on nest.** **A,** Imidacloprid, Week 0: F = 2.55, DF = 4, 35, p = 0.057, Week 2: F = 4.20, DF = 4, 17, p = 0.016, Week 4: F = 4.82, DF = 4, 16, p = 0.010, Week 6: F = 3.84, DF = 4, 12, p = 0.031, Week 8: F = 1.77, DF = 3, 17, p = 0.192. **B,** Clothianidin, Week 0: F = 0.39, DF = 4, 37, p = 0.813, Week 2: F = 0.21, DF = 4, 36, p = 0.928, Week 4: F = 2.16, DF = 4, 33, p = 0.095, Week 6: F = 4.52, DF = 4, 28, p = 0.006, Week 8: F = 8.29, DF = 4, 8, p = 0.005. ANOVA, Tukey-Kramer MRT by treatment for each week are on the figures to compare the 2 chemicals, but ProcMixed did not show a significant interaction for imidacloprid, but did for clothianidin, (Table S1).
(TIF)

Table S1 **Statistical analysis.** When a week effect in ProcMixed is significant, the Tukey-Kramer MRT is on the figure and the statistics are on this table. When a treatment effect in ProcMixed is significant, the statistics, mean, SE, and Tukey-Kramer MRT for each treatment is on this table (SAS, 2010). When an interaction effect is significant in ProcMixed, the statistics are on this table. Then the data were analyzed individually by week for treatment and the statistics are on the figure legend (ANOVA, Tukey-Kramer MRT, SAS, JMP, 2010).
(DOCX)

Table S2 **Individual bee consumption in ml and ng by treatment for each week.** Imidacloprid, Week 2: F = 30.97, DF = 4, 16, p<0.001, Week 4: F = 10.31, DF = 4, 33, p<0.001, Week 6: F = 0.89, DF = 4, 8, p = 0.513, Week 8: F = 2.51, DF = 3, 17, p = 0.093, Clothianidin, Week 2: F = 17.68, DF = 4, 17, p<0.001, Week 4: F = 32.73, DF = 4, 15, p<0.001, Week 6: F = 9.37, DF = 4, 28, p<0.001, Week 8: F = 4.32, DF = 4, 8, p = 0.035, ANOVA, Tukey-Kramer MRT by treatment for each week.
(DOCX)
