# Supplementary figures and images for: Chronic Exposure of Imidacloprid and Clothianidin Reduce Queen Survival, Foraging, and Nectar Storing in Colonies of Bombus impatiens
Source: PLoS One. 2014 Mar 18;9(3):e91573. doi: 10.1371/journal.pone.0091573 (PMC3958374; doi:10.1371/journal.pone.0091573)

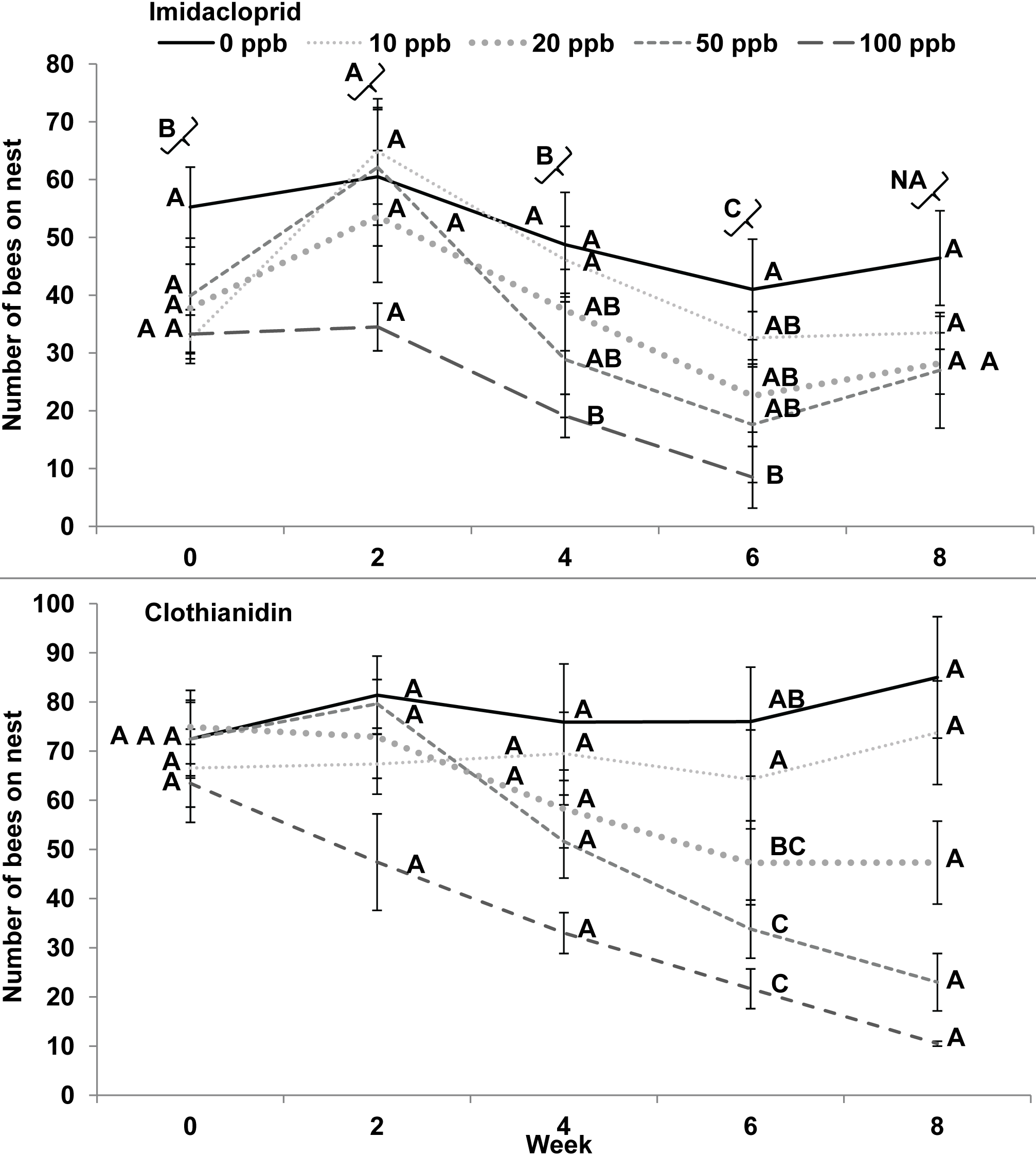

Supplement: Figure S1 — Bees on nest. A, Imidacloprid, Week 0: F = 2.55, DF = 4, 35, p = 0.057, Week 2: F = 4.20, DF = 4, 17, p = 0.016, Week 4: F = 4.82, DF = 4, 16, p = 0.010, Week 6: F = 3.84, DF = 4, 12, p = 0.031, Week 8: F = 1.77, DF = 3, 17, p = 0.192. B, Clothianidin, Week 0: F = 0.39, DF = 4, 37, p = 0.813, Week 2: F = 0.21, DF = 4, 36, p = 0.928, Week 4: F = 2.16, DF = 4, 33, p = 0.095, Week 6: F = 4.52, DF = 4, 28, p = 0.006, Week 8: F = 8.29, DF = 4, 8, p = 0.005. ANOVA, Tukey-Kramer MRT by treatment for each week are on the figures to compare the 2 chemicals, but ProcMixed did not show a significant interaction for imidacloprid, but did for clothianidin, (Table S1). (TIF) [file pone.0091573.s001.tif]
