# Supplementary material for: Chronic Exposure of Imidacloprid and Clothianidin Reduce Queen Survival, Foraging, and Nectar Storing in Colonies of Bombus impatiens
Source: PLoS One. 2014 Mar 18;9(3):e91573. doi: 10.1371/journal.pone.0091573 (PMC3958374; doi:10.1371/journal.pone.0091573)
Supplement: Table S1 — Statistical analysis. When a week effect in ProcMixed is significant, the Tukey-Kramer MRT is on the figure and the statistics are on this table. When a treatment effect in ProcMixed is significant, the statistics, mean, SE, and Tukey-Kramer MRT for each treatment is on this table (SAS, 2010). When an interaction effect is significant in ProcMixed, the statistics are on this table. Then the data were analyzed individually by week for treatment and the statistics are on the figure legend (ANOVA, Tukey-Kramer MRT, SAS, JMP, 2010). (DOCX) [file pone.0091573.s002.docx]

| **Table S1:** Statistical analysis. When a week effect in ProcMixed is significant, the Tukey-Kramer MRT is on the figure and the statistics are on this table. When a treatment effect in ProcMixed is significant, the statistics, mean, SE, and Tukey-Kramer MRT for each treatment is on this table (SAS, 2010). When an interaction effect is significant in ProcMixed, the statistics are on this table. Then the data were analyzed individually by week for treatment and the statistics are on the figure legend (ANOVA, Tukey-Kramer MRT, SAS, JMP, 2010). | | | | | |
| --- | --- | --- | --- | --- | --- |
| **Figure** | **Parameter** | **Wk/trt** | **Week effect F (df), P** | **Treatment effectF (df), P** | **Interaction effectF (df), P** |
| **imidacloprid** | | | | | |
| **2** | **Colony consumption** | 2, 4, 6, 8 | 1.91 (3,77), 0.1356 | 32.4 (4,35), 0.0001 | 2.35 (11,77), 0.0148 |
|  |  | Trt (ppb) | n | Mean ±SE | Tukey-Kramer |
|  |  | 0 | 31 | 0.78±0.05 | A |
|  |  | 10 | 32 | 0.50±0.05 | B |
|  |  | 20 | 27 | 0.30±0.05 | C |
|  |  | 50 | 21 | 0.15±0.06 | C |
|  |  | 100 | 20 | - | - |
| **3** | **Bee Consumption**week effect on figure | 2, 4, 6, 8 | 8.52 (3,76), 0.0001 | 1.59 (4,35), 0.1998 | 0.87 (11,76), 0.5698 |
|  |  | Trt (ppb) | n | Mean ±SE | Tukey-Kramer |
|  |  | 0 | 31 | 1.56±0.23 | A |
|  |  | 10 | 32 | 1.13±0.25 | A |
|  |  | 20 | 27 | 0.91±0.23 | A |
|  |  | 50 | 21 | 0.61±0.37 | A |
|  |  | 100 | 19 | - | - |
| **S1** | **Bees on nest** week effect on figure | 0, 2, 4, 6, 8 | 21.4(4,112), 0.0001 | 3.67 (4,35), 0.0135 | 1.34 (15,112), 0.1910 |
|  |  | Trt (ppb) | n | Mean ±SE | Tukey-Kramer |
|  |  | 0 | 39 | 50.49±10.05 | A |
|  |  | 10 | 40 | 41.90±9.39 | A |
|  |  | 20 | 35 | 36.91±7.60 | A |
|  |  | 50 | 29 | 39.07±8.37 | A |
|  |  | 100 | 28 | 26.04±6.83 | - |
| **Resultssection** | **Bee weight** | 4, 6, 8 | 8.76 (2,38), 0.0007 | 2.20 (4,35), 0.0894 | 0.41 (8,38), 0.9096 |
|  |  | Trt (ppb) | n | Mean±SE | Tukey-Kramer |
|  |  | 0 | 21 | 0.14±0.0083 | A |
|  |  | 10 | 21 | 0.14±0.0083 | A |
|  |  | 20 | 16 | 0.12±0.0095 | A |
|  |  | 50 | 16 | 0.12±0.0095 | A |
|  |  | 100 | 14 | 0.11±0.010 | A |
| **clothianidin** | | | | | |
| **2** | **Colony consumption** | 2, 4, 6, 8 | 1.72 (3,85), 0.1689 | 85.7 (4,36) 0.0001 | 2.76 (12,85), 0.0032 |
|  |  | Trt (ppb) | n | Mean±SE | Tukey-Kramer |
|  |  | 0 | 36 | 84.21±3.27 | A |
|  |  | 10 | 32 | 58.81±3.47 | B |
|  |  | 20 | 31 | 26.30±3.50 | C |
|  |  | 50 | 23 | 10.46±3.87 | D |
|  |  | 100 | 19 | 7.04±4.29 | D |
| **3** | **Bee consumption, week effect on figure** | 2, 4, 6, 8 | 3.53 (3,84), 0.0183 | 14.13(4,36), 0.0001 | 0.96 (12,84), 0.4918 |
|  |  | Trt (ppb) | n | Mean ± SE | Tukey-Kramer |
|  |  | 0 | 36 | 1.13±0.094 | A |
|  |  | 10 | 31 | 0.73±0.10 | B |
|  |  | 20 | 31 | 0.44±0.10 | BC |
|  |  | 50 | 23 | 0.25±0.11 | C |
|  |  | 100 | 19 | 0.19±0.12 | C |
| **S1** | **Bees on nestweek effect on figure** | 0, 2, 4, 6, 8 | 26.9(4,120), 0.0001 | 2.95 (4,37), 0.0328 | 3.99(16,120), 0.0001 |
|  |  | Trt (ppb) | n | Mean±SE | Tukey-Kramer |
|  |  | 0 | 45 | 69.96±6.62 | A |
|  |  | 10 | 39 | 70.45±7.04 | A |
|  |  | 20 | 40 | 55.56±6.79 | A |
|  |  | 50 | 31 | 47.78±7.37 | A |
|  |  | 100 | 27 | 43.77±7.75 | A |
| **Resultssection** | **Bee weight** | 4, 6, 8 | 4.53 (2,46), 0.0161 | 5.58 (4,34), 0.0015 | 1.96 (7,46), 0.0807 |
|  |  | Trt (ppb) | n | Mean±SE | Tukey-Kramer |
|  |  | 0 | 27 | 0.12±0.69 | A |
|  |  | 10 | 24 | 0.13±0.75 | AB |
|  |  | 20 | 23 | 0.15±0.91 | B |
|  |  | 50 | 13 | 0.16±0.70 | AB |
|  |  | 100 | 7 | 0.11±0.56 | - |
