# Supplementary material for: Chronic Exposure of Imidacloprid and Clothianidin Reduce Queen Survival, Foraging, and Nectar Storing in Colonies of Bombus impatiens
Source: PLoS One. 2014 Mar 18;9(3):e91573. doi: 10.1371/journal.pone.0091573 (PMC3958374; doi:10.1371/journal.pone.0091573)
Supplement: Table S2 — Individual bee consumption in ml and ng by treatment for each week. Imidacloprid, Week 2: F = 30.97, DF = 4, 16, p<0.001, Week 4: F = 10.31, DF = 4, 33, p<0.001, Week 6: F = 0.89, DF = 4, 8, p = 0.513, Week 8: F = 2.51, DF = 3, 17, p = 0.093, Clothianidin, Week 2: F = 17.68, DF = 4, 17, p<0.001, Week 4: F = 32.73, DF = 4, 15, p<0.001, Week 6: F = 9.37, DF = 4, 28, p<0.001, Week 8: F = 4.32, DF = 4, 8, p = 0.035, ANOVA, Tukey-Kramer MRT by treatment for each week. (DOCX) [file pone.0091573.s003.docx]

| **Table S2.** Individual bee consumption in ml and ng by treatment for each week. Imidacloprid, Week 2: F = 30.97, DF = 4, 16, p<0.001, Week 4: F = 10.31, DF = 4, 33, p<0.001, Week 6: F = 0.89, DF = 4, 8, p = 0.513, Week 8: F = 2.51, DF = 3, 17, p = 0.093, Clothianidin, Week 2: F = 17.68, DF = 4, 17, p<0.001, Week 4: F = 32.73, DF = 4, 15, p<0.001, Week 6: F = 9.37, DF = 4, 28, p<0.001, Week 8: F = 4.32, DF = 4, 8, p = 0.035, ANOVA, Tukey-Kramer MRT by treatment for each week | | | | | | |
| --- | --- | --- | --- | --- | --- | --- |
| **Imidacloprid trt*** | **week** | **No bees** | **No colonies** | **mean ml consumed** | **± SE** | **ng consumed**trt (residue)** |
| 0 (0) | 2 | 61 | 8 | 1.42999 | 0.25893 | 0 (0) |
| 0 (0)0 | 4 | 49 | 8 | 1.24777 | 0.14967 | 0 (0) |
| 0 (0) | 6 | 41 | 8 | 2.09384 | 0.6222 | 0 (0) |
| 0 (0)0 | 8 | 46 | 7 | 1.46522 | 0.22418 | 0 (0) |
| 10 (14) | 2 | 65 | 8 | 0.70978 | 0.05295 | 7.1 (9.9) |
| 10 (14) | 4 | 46 | 8 | 1.01269 | 0.06280 | 10.1 (14.1) |
| 10 (14) | 6 | 33 | 8 | 1.00837 | 0.1705 | 10.1 (14.0) |
| 10 (14) | 8 | 34 | 8 | 0.89297 | 0.17523 | 8.9 (11.1) |
| 20 (16) | 2 | 54 | 8 | 0.48825 | 0.06616 | 9.6 (7.7) |
| 20 (16) | 4 | 37 | 7 | 0.74858 | 0.10279 | 15.0 (11.8) |
| 20 (16) | 6 | 23 | 7 | 2.23146 | 1.3026 | 4.5 (35.7) |
| 20 (16) | 8 | 28 | 5 | 0.94857 | 0.20027 | 19.0 (15.0) |
| 50 (71) | 2 | 62 | 8 | 0.16106 | 0.01713 | 8 .0 (11.4) |
| 50 (71) | 4 | 29 | 7 | 0.36550 | 0.05838 | 18.3 (25.6) |
| 50 (71) | 6 | 18 | 5 | 1.56840 | 0.7354 | 7.8 (110.8) |
| 50 (71) | 8 | 27 | 1 | 0.27160 | - | 13.6 (19.2) |
| 100 (127) | 2 | 35 | 8 | 0.17845 | 0.02197 | 17.8 (21.6) |
| 100 (127) | 4 | 19 | 8 | 0.47517 | 0.14935 | 47.5 (59.7) |
| 100 (127) | 6 | 9 | 3 | 2.72029 | 2.3082 | 272.0 (345.4) |
| 100 (127) | 8 | 0 | 0 | - | - | - |
| **Clothianidin trt*** | **week** | **No bees** | **No colonies** | **mean ml consumed** | **± SE** | **ng consumed**trt (residue)** |
| 0 (0) | 2 | 67 | 9 | 0.919682 | 0.14505 | 0 (0) |
| 0 (0) | 4 | 75 | 9 | 0.969030 | 0.11866 | 0 (0) |
| 0 (0) | 6 | 72 | 9 | 1.06257 | 0.11362 | 0 (0) |
| 0 (0) | 8 | 63 | 9 | 1.56736 | 0.38544 | 0 (0) |
| 10 (9) | 2 | 67 | 8 | 0.611672 | 0.05839 | 6.1 (5.5) |
| 10 (9) | 4 | 73 | 8 | 0.704457 | 0.06912 | 7.0 (6.3) |
| 10 (9) | 6 | 79 | 8 | 0.68567 | 0.12723 | 6.9 (6.1) |
| 10 (9) | 8 | 47 | 7 | 0.93701 | 0.20287 | 9.4 (8.4) |
| 20 (17) | 2 | 70 | 8 | 0.354740 | 0.06508 | 7.0 (6.0) |
| 20 (17) | 4 | 58 | 8 | 0.473250 | 0.08005 | 9.5 (8.0) |
| 20 (17) | 6 | 52 | 8 | 0.43893 | 0.05931 | 8.8 (7.3) |
| 20 (17) | 8 | 33 | 7 | 0.50118 | 0.07208 | 10.0 (8.5) |
| 50 (39) | 2 | 64 | 8 | 0.185079 | 0.02668 | 9.0 (7.0) |
| 50 (39) | 4 | 47 | 7 | 0.211047 | 0.02728 | 10.6 (8.2) |
| 50 (39) | 6 | 34 | 5 | 0.30530 | 0.12282 | 15.3 (11.7) |
| 50 (39) | 8 | 22 | 3 | 0.32934 | 0.13535 | 16.5 (12.5) |
| 100 (76) | 2 | 74 | 8 | 0.154299 | 0.02834 | 15 (11.4) |
| 100 (76) | 4 | 47 | 6 | 0.102446 | 0.01239 | 10.2 (7.6) |
| 100 (76) | 6 | 23 | 3 | 0.17881 | 0.04060 | 17.8 (12.9) |
| 100 (76) | 8 | 11 | 2 | 0.31970 | 0.04697 | 31.9 (23.6) |

* Treatment values in parentheses indicate the residue from analysis, ** 10 ppb = 10ng/ml= 10 x ml consumed
